# Fine control for the preparation of ceria nanorods (111)†

**DOI:** 10.1039/d3ra02817h

**Published:** 2023-07-14

**Authors:** Changju Yang, Xiang Ning, Shanyong Chen, Xiaoxia Hou, Xiaoli Xia, Zhiyang Zhang, Weiping Ding, Luming Peng

**Affiliations:** a Key Laboratory of Mesoscopic Chemistry of MOE, School of Chemistry and Chemical Engineering, Nanjing University Nanjing 210023 China luming@nju.edu.cn

## Abstract

The morphologies and exposed surfaces of ceria nanocrystals are important factors in determining their performance. In order to establish a structure–property relationship for ceria nanomaterials, it is essential to have materials with well-defined morphologies and specific exposed facets. This is also crucial for acquiring high resolution ^17^O solid-state NMR spectra. In this study, we explore the synthesis conditions for preparing CeO_2_ nanorods with exposed (111) facets. The effects of alkali concentration, hydrothermal temperature and time, cerium source and oxidation agent are investigated and optimal synthesis conditions are found. The resulting CeO_2_ nanorods show very narrow ^17^O NMR peaks for the oxygen ions in the first, second and third layers, providing a foundation for future research on mechanisms involving ceria materials using ^17^O solid-state NMR spectroscopy.

## Abstract 2

The synthesis of CeO_2_ nanorods with exposed (111) facets is optimized and the obtained material shows very narrow ^17^O NMR peaks, providing a foundation for future research on mechanisms using ^17^O solid-state NMR spectroscopy.

## Graphical abstract

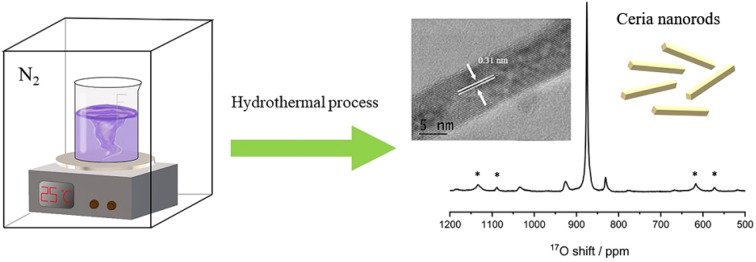

## Introduction

CeO_2_, a rare-earth metal oxide with cubic fluorite structure, has many attractive properties and is critical in the chemical industry.^1–5^ It is particularly useful for providing oxygen in oxygen-deficient environments, generating nonstoichiometric oxide CeO_2−*x*_, while this reduced oxide can store oxygen under oxygen-rich conditions.^6^ Therefore, CeO_2_ finds multiple applications in redox catalysis, including as three-way catalysts (TWCs),^7^ in the water gas conversion reaction (WGS),^8^ oxidation of volatile organic matter,^9^ hydrogen purification,^10^ petroleum cracking,^11^ CO_2_ hydrogenation,^12^ and as a catalyst support.^13^ Its excellent properties can be ascribed to its special geometric structure^14^ and electronic structure,^15^ as well as the low activation energy barrier for generating lattice oxygen vacancies.

Nanomaterials, which have attracted a lot of research attention recently, are often associated with better catalytic properties. It has been found that the morphologies^16^ and exposed facets of nanocrystals play a crucial role in controlling the catalytic activity and selectivity.^17–20^ In order to explore the relationship between structure and catalytic properties in detail, nanomaterials with well-defined morphologies and specific exposed facets are required. Many attempts have been made to prepare CeO_2_ nanocrystals with specific morphology and facets,^21–23^ and it is often necessary to control the synthesis conditions, including alkali concentration, hydrothermal temperature and anions in the cerium source.^24,25^

We have recently developed a new method based on ^17^O solid-state NMR spectroscopy for distinguishing oxygen ions in different surface layers or different facets in oxide nanomaterials according to the chemical shift.^26,27^ This method can be used to explore the detailed reaction mechanisms on these materials. The linewidths of the signals are dependent on the distribution of the local environments, such as bond angles and bond length. In order to obtain high resolution data and detailed structural information, the peak widths should be minimized. Therefore, CeO_2_ nanocrystals with specific morphology and facets are required. In this paper, we explore the template-free hydrothermal synthesis of CeO_2_ nanomaterials, and prepare CeO_2_ nanorods exposing mainly (111) facets by controlling the alkaline solution concentration, hydrothermal temperature and time, cerium source and oxidation agent. We show that the CeO_2_ nanorods prepared under optimized conditions exhibit very narrow linewidths in the ^17^O NMR spectrum.

## Experimental section

### Synthesis

CeO_2_ nanoparticles with different morphologies were synthesized using a hydrothermal method. Solutions of Ce(NH_4_)_2_(NO_3_)_6_, CeCl_3_ and Ce(NO_3_)_3_ were prepared with a concentration of 0.05 mol L^−1^ to serve as sources of cerium ions. NaOH solutions with different concentrations (0.1, 1, 3 and 6 mol L^−1^) were also prepared. Deionized water was used as the solvent in all the above solutions. To synthesize the CeO_2_ nanoparticles, the solution containing Ce ions was mixed with the NaOH solution at a volume ratio of 1 : 7 under high-speed stirring at room temperature. The resulting mixture was stirred for 30 min and then transferred to a hydrothermal reactor for heating at 100 to 180 °C for 24 h. After the hydrothermal treatment, the product was filtered, washed with deionized water and ethanol until a neutral pH was obtained, and then heated in an oven at 80 °C for 3 h. Finally, the product was calcined at 700 °C for 3 h under an air atmosphere, with the calcination temperature determined using thermogravimetric analysis (TGA) data (Fig. S1†). Further details on the preparation procedures are discussed in the Results and discussion section.

### Characterization

X-ray diffraction (XRD) was carried out on a Philips X'pert Pro diffractometer using Kα radiation from a Cu target (*λ* = 0.15418 nm) with a Ni filter. The operating current and voltage were 40 mA and 40 kV, respectively. The scanning range of 2*θ* was from 5° to 90°. Transmission electron microscopy (TEM) images of samples were taken on a JEOL-JEM-2010 transmission electron microscope operating at 100 kV. The TGA was carried out on a NETZSCH STA 449C, from room temperature to 700 °C. The BET surface area and pore size distribution were determined from nitrogen isotherm at 77 K on a Micromeritics TriStar II 3020 instrument. X-ray photoelectron spectroscopy (XPS) was performed using a PHI 5000 Versa Probe manufactured by ULVAC-PHI, Japan.

^17^O magic angle spinning (MAS) nuclear magnetic resonance (NMR) experiments were performed on a Bruker Avance III 400 MHz solid-state NMR spectrometer using 4.0 mm MAS probes tuned to ^17^O at 54.2 MHz. ^17^O chemical shifts are referenced to H_2_O at 0.0 ppm. A short excitation pulse of 1.2 μs, corresponding to π/6 pulse for H_2_O, and a recycle delay of 5 s were used. Prior to NMR experiments, the CeO_2_ sample was placed in a glass tube, heated at 300 °C and exposed to vacuum for 12 h, before it was exposed to O_2_ (Sigma-Aldrich, 90% ^17^O) and further heated at 300 °C for 12 h. The sample was packed in zirconia MAS NMR rotor in a N_2_-filled glove box and spun at 14 kHz.

## Results and discussion

**Fig. 1:**
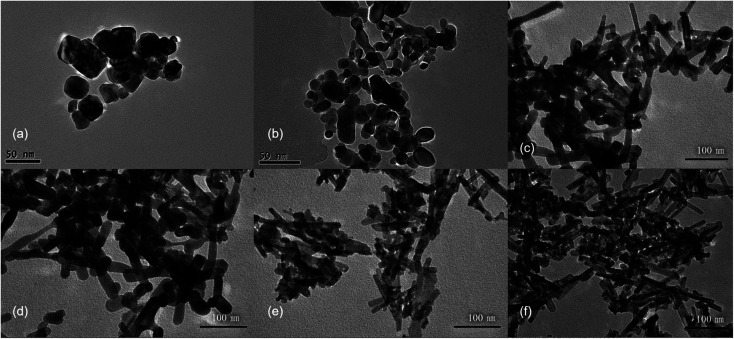
The TEM images of CeO_2_ nanostructures obtained by pouring 0.05 mol L^−1^ CeCl_3_ solution to NaOH solutions with different concentrations. (a) 0.1 mol L^−1^, (b) 1 mol L^−1^, (c) 3 mol L^−1^, (d) 4 mol L^−1^, (e) 5 mol L^−1^ and (f) 6 mol L^−1^. Hydrothermal temperature: 100 °C, time: 24 h.

First, CeCl_3_ solution was poured into NaOH solutions at different concentrations to prepare CeO_2_ nanostructures. The XRD patterns (Fig. S2†) show characteristic diffraction peaks for CeO_2_ (JCPDS No. 34-0394). The relatively broad widths of the diffraction peaks, based on the Debye–Scherrer equation, suggests that the particle sizes should be relatively small. In addition, the widths of the diffraction peaks in XRD are similar for different samples, indicating that the sizes of different samples are similar. Transmission electron microscopy (TEM) was used to further characterize the CeO_2_ nanoparticles (Fig. 1), revealing that morphologies of different products are quite different, despite the similar sizes based on the XRD data. CeO_2_ samples generated with a low NaOH concentration of 0.1 to 1 mol L^−1^, are mainly irregular particles with sizes ranging from 30 to 50 nm (Fig. 1a and b). With increasing NaOH concentration (3 to 5 mol L^−1^), the products become rod-like, however, there is still a considerable proportion of nanoparticles in the product (Fig. 1c–e). When the concentration of the NaOH solution reaches 6 mol L^−1^, nanorods dominate with an average diameter of 10 nm, while a small number of nanoparticles also exists. These results indicate that a more concentrated alkali solution leads to more rapid dissolution/recrystallization rate of Ce(OH)_3_, and thus the rod-like morphology of CeO_2_.^28^

**Fig. 2:**
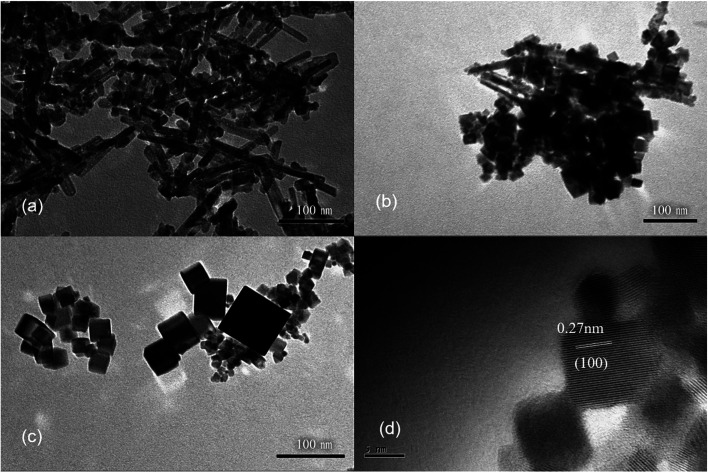
The TEM images of CeO_2_ nanostructures obtained with different hydrothermal temperatures. (a) 100 °C, (b) 140 °C and (c) 180 °C. (d) The HRTEM image of CeO_2_ nanostructures shown in (c). Hydrothermal time: 24 h. Cerium source: 0.05 mol L^−1^ CeCl_3_ solution. NaOH solution concentration: 6 mol L^−1^.

To investigate the effects of hydrothermal temperature on the morphology, the alkali solution with a concentration of 6 mol L^−1^ was used, and three hydrothermal temperatures (100, 140 and 180 °C) were employed to prepare CeO_2_ nanostructures. The XRD patterns (Fig. S3†) confirm that the obtained products are pure CeO_2_. Despite the similarity of the XRD patterns, the TEM images of the three products are very different (Fig. 2). At a low hydrothermal temperature of 100 °C, nanorods with a diameter of 10 nm and some nanoparticles are obtained, while at a higher hydrothermal temperature of 140 °C, many nanocubes with a length of 25–40 nm show up in the products, along with fewer nanorods. Most of products are nanocubes with a slightly larger size of 25–50 nm at a hydrothermal temperature of 180 °C, and the HRTEM image show that these nanocubes mainly exposes (100) surface. Therefore, a low hydrothermal temperature of 100 °C is found to be very important to obtain nanorods exposing (111) facets.

**Fig. 3:**
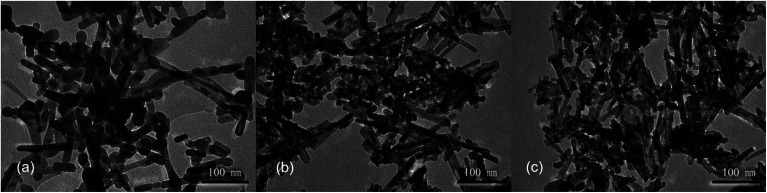
The TEM images of CeO_2_ nanostructures obtained with different hydrothermal time. (a) 12 h, (b) 24 h and (c) 36 h. Hydrothermal temperature: 100 °C. Cerium source: 0.05 mol L^−1^ CeCl_3_ solution. NaOH solution concentration: 6 mol L^−1^.

Next, hydrothermal treatment time was further optimized at a hydrothermal temperature of 100 °C using the alkali solution with a concentration of 6 mol L^−1^. Again, similar XRD patterns are observed for the products obtained with different hydrothermal times of 12, 24 and 36 h, confirming the formation of CeO_2_ phases (Fig. S4†). With a hydrothermal treatment time of 12 h, the product contains both nanorods and nanoparticles (Fig. 3a). With a longer hydrothermal time of 24 h, the amount of nanoparticles decreases and nanorods dominate, while the products are similar at a longer hydrothermal time of 36 h (Fig. 3b and c). These results suggest that a relatively long hydrothermal time of 24 h is required for the nanorods to form and thus 24 h is chosen as the optimized hydrothermal time.

**Fig. 4:**
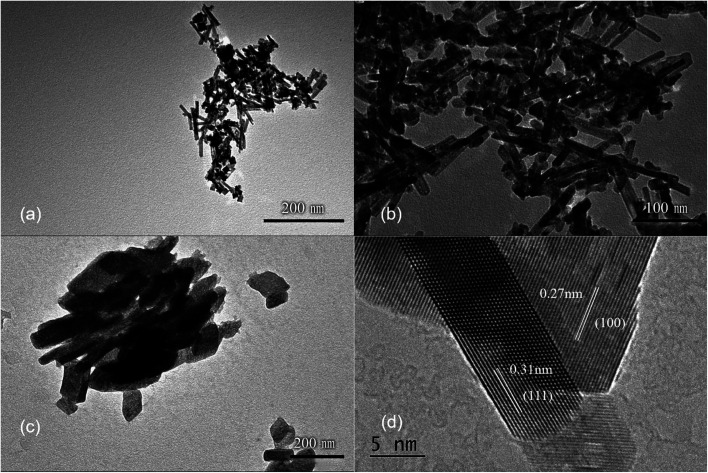
The TEM images of CeO_2_ nanostructures obtained with different cerium sources. (a) Ce(NO_3_)_3_, (b) CeCl_3_, (c) Ce(NH_4_)_2_(NO_3_)_6_. (d) The HRTEM image of CeO_2_ nanostructures shown in (a). Hydrothermal temperature and time: 100 °C and 24 h. NaOH solution concentration: 6 mol L^−1^.

It has been shown that the morphology of CeO_2_ nanoparticles may also be related to the Ce salts used present in the synthesis,^29^ therefore, different cerium sources were also tested toward the synthesis of CeO_2_ nanorods. Three common cerium compounds, including Ce(NH_4_)_2_(NO_3_)_6_, CeCl_3_ and Ce(NO_3_)_3_, which have different oxidation states for Ce, were selected for the synthesis. The XRD patterns (Fig. S5†) show that CeO_2_ phases can be obtained in all three cases. However, different morphologies are observed for the products. The TEM images show that nanorods dominate in the products, when using Ce(NO_3_)_3_ or CeCl_3_ as the cerium source (Fig. 4a and b). The CeO_2_ nanorods obtained have a diameter of approximately 10 nm in both cases, while the length of the nanorods is 100–200 nm with Ce(NO_3_)_3_ as the source and the length decreases to 20–50 nm if CeCl_3_ is used. However, when using Ce(NO_3_)_3_ as the cerium source, a small amount of nanocubes is attached to the nanorods, as shown by HRTEM (Fig. 4d). Because NO_3_^−^ is more inclined to be adsorbed on (100) surface, it is conducive to the growth of nanocubes.^29,30^ In contrast, larger nanosheets are produced when Ce(NH_4_)_2_(NO_3_)_6_ is used as the starting material (Fig. 4c).

The differences observed in the products by using Ce^3+^ or Ce^4+^ salts as the source should be related to the formation mechanism of CeO_2_. Ce(OH)_3_ is first formed rapidly with Ce^3+^ salts as the starting materials, while it is further oxidized to form CeO_2_, which is expected to be slower. No oxidation process is required if Ce(NH_4_)_2_(NO_3_)_6_ is used as the cerium source, leading to a much faster process.^31^ Therefore, the slow oxidation in the hydrothermal process is expected to play a key role in the formation of CeO_2_ in a nanorod morphology.

**Fig. 5:**
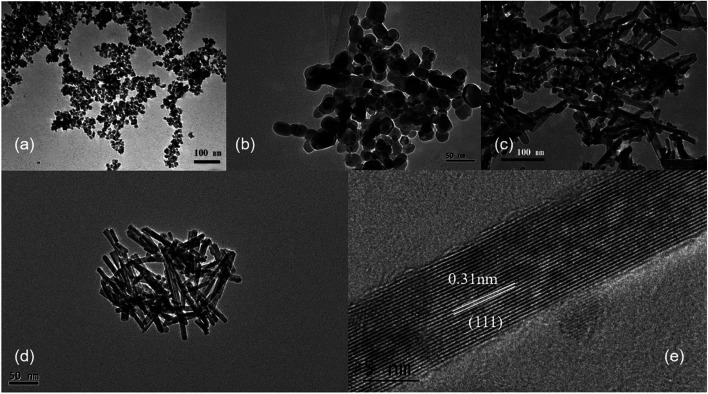
The TEM images of CeO_2_ nanostructures obtained with different oxidants. (a) H_2_O_2_, (b) air (reaction time: 120 min), (c) air (reaction time: 30 min) and (d) trace oxygen in N_2_ atmosphere (reaction time: 30 min). (e) The HRTEM image of CeO_2_ nanorods shown in (d). Hydrothermal temperature and time: 100 °C and 24 h. Cerium source: 0.05 mol L^−1^ CeCl_3_ solution. NaOH solution concentration: 6 mol L^−1^.

Ce^3+^ can be oxidized by a variety of methods, such as roasting oxidation, electrolytic oxidation, chemical oxidation and gas oxidation (such as oxygen gas or air).^32^ Here we investigated the effects using different chemical reagents or oxygen gas concentrations. The XRD patterns of the corresponding samples are shown in Fig. S6,† confirming the formation of CeO_2_ phases in all four cases. When H_2_O_2_ was used as the chemical oxidant, it was added to the suspension generated after quickly mixing CeCl_3_ and NaOH solution. Granular and relatively uniform nanoparticles with small particle sizes of approximately 15 nm are obtained (Fig. 5a). In order to reduce the oxidation rate, air was used as the oxidant, while a constant-flow pump was used to slowly drop the CeCl_3_ solution into the NaOH solution under vigorous stirring, in order to control the formation rate of Ce(OH)_3_ as well as oxidation, and the process was completed in 120 min. Again, the obtained products are granular and do not have specific shapes (Fig. 5b). Sample agglomeration also occurs, forming relatively large particles with a diameter of more than 50 nm, indicating that the reaction time for generating the precursor (Ce(OH)_3_) as well as the oxidation process is too long. By increasing the rate for dropping CeCl_3_ solution, the total reaction time can be decreased to 30 min, resulting in nanorod products with a small number of particles (Fig. 5c). By applying N_2_ atmosphere protection and fixing the reaction time to 30 min, the obtained samples are all rod-shaped (Fig. 5d). The average diameter is as small as 10 nm and the length is about 100–200 nm, which is associated with a large specific surface area (115 m^2^ g^−1^). The results suggest that very low oxygen pressure (trace oxygen in nitrogen environment) is one of the keys in preparing CeO_2_ nanorods.^33^ The reaction time for forming the precursor (Ce(OH)_3_), on the other hand, should be limited (*i.e.*, adding CeCl_3_ solution to NaOH solution within 30 minutes) in order to avoid agglomeration. The HRTEM image of the same sample shown in Fig. 5d exhibits that the exposed surface of is (111), the most energetically favorable facet for CeO_2_ (Fig. 5e). This nanorod sample with the most uniform morphology exposing (111) facet is named as CeO_2_-NR(111).

**Fig. 6:**
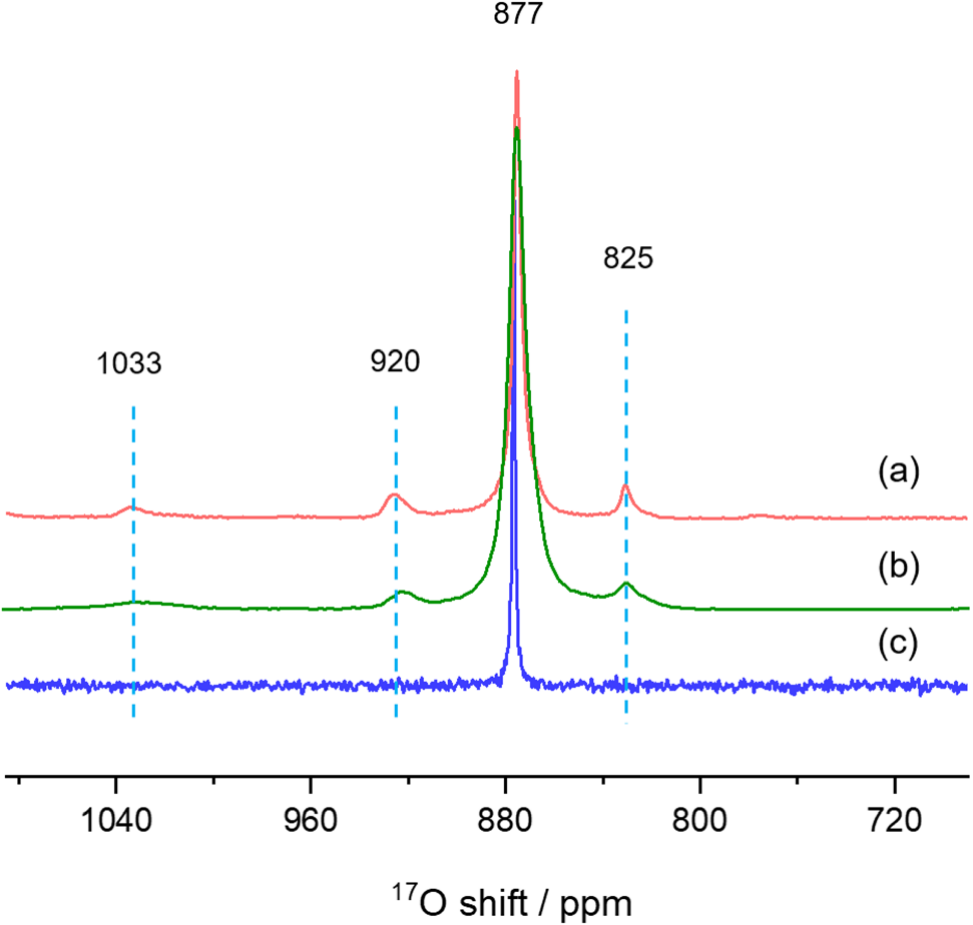
^17^O MAS NMR spectra of (a) CeO_2_-NR(111), (b) CeO_2_-L and (c) bulk ceria commercially available.

Finally, we used solid-state NMR spectroscopy to study the local environments of oxygen in the CeO_2_-NR(111) sample, and compared the results to the spectrum of CeO_2_ nanorods prepared by literature method. To facilitate the comparison, the ^17^O NMR spectrum of commercially available micron-sized ceria was collected (Fig. 6c),^26^ which shows a sharp peak at 877 ppm, arising from the 4-coordinated oxygen ions (OCe_4_) in the bulk part of ceria. The ^17^O MAS NMR spectrum of CeO_2_-NR(111) enriched with ^17^O_2_ at 300 °C (Fig. 6a) shows four peaks at 1033, 920, 825 and 877 ppm, which can be assigned to the oxygen ions at the first, second, third layers of ceria (111) facets, and OCe_4_ in the bulk part of the nanostructure, respectively, according to the previous work,^26^ which confirms the successful preparation of CeO_2_ nanorods preferentially exposing (111) facets. CeO_2_ nanorods synthesized according to the method used in our previous ^17^O NMR paper (CeO_2_-L) show four peaks at 1033, 920, 825 and 877 ppm in the ^17^O MAS NMR spectrum (Fig. 6b) and the frequencies are similar to CeO_2_-NR(111).^20,34^ However, the peaks of CeO_2_-L are much broader than CeO_2_-NR(111) (Table 1), indicating that CeO_2_-NR(111) has a more ordered surface structure.^35^ Therefore, CeO_2_-NR(111) is a better material suitable for ^17^O NMR studies of ceria nanorods. It is worth mentioning that the additional peaks at 1033, 920 and 825 ppm due to oxygen ions at first, second and third layers are not observed in micron-sized ceria (Fig. 6c), which can be ascribed to the very small surface area and the corresponding low concentrations of these surface sites in this sample.

**Table 1:** Comparison of the full width at half maximum (FWHM) of the peaks in ^17^O MAS NMR spectrum of CeO_2_-NR(111) and CeO_2_-L

Chemical shift/ppm	FWHM(CeO_2_-NR(111))/ppm	FWHM(CeO_2_-L)/ppm
831	5.3	17.1
877	3.8	8.2
926	10.9	16.6
1034	14.3	38.8

## Conclusion

In this study, we explored the hydrothermal synthesis conditions for preparing CeO_2_ nanorods that exposes (111) facets with a well-defined structure suitable for ^17^O solid state NMR investigations. Several important factors affecting the morphology of CeO_2_ nanostructures were tested, including alkali solution concentration, hydrothermal temperature and time, cerium source and oxidation agent. The CeO_2_ nanorods prepared with the optimized conditions exhibit much narrower signals in the ^17^O MAS NMR spectrum, compared to the those prepared using the method from previous work. This study provides a foundation for future investigations into detailed mechanisms of reactions involving CeO_2_ nanorods (111) using ^17^O solid state NMR spectroscopy.

## Author contributions

C. Yang, L. Peng and W. Ding, contributed the design of the experiment and writing of the paper. X. Ning, X. Hou, and X. Xia carried out characterization of the samples. S. Chen and Z. Zhang took the TEM and HRTEM images of the samples. All the authors have given their approval to the final version of the manuscript.

## Conflicts of interest

The authors declare no competing interests.

## Supplementary Material

RA-013-D3RA02817H-s001
