# Supplementary material for: Fine control for the preparation of ceria nanorods (111)
Source: RSC Adv. 2023 Jul 14;13(31):21271–6. doi: 10.1039/d3ra02817h (PMC10346444; doi:10.1039/d3ra02817h)
Supplement: RA-013-D3RA02817H-s001 [file RA-013-D3RA02817H-s001.pdf]

Electronic Supplementary Material (ESI) for RSC Advances.
This journal is © The Royal Society of Chemistry 2023

Supplementary Information

for

Fine Control for the Preparation of Ceria Nanorods (111)

Changju Yang, Xiang Ning, Shanyong Chen, Xiaoxia Hou, Xiaoli Xia, Zhiyang Zhang, Weiping Ding, Luming Peng*

Key Laboratory of Mesoscopic Chemistry of MOE, School of Chemistry and Chemical Engineering, Nanjing University, Nanjing 210023, China

*Corresponding Author: Luming Peng: luming@nju.edu.cn

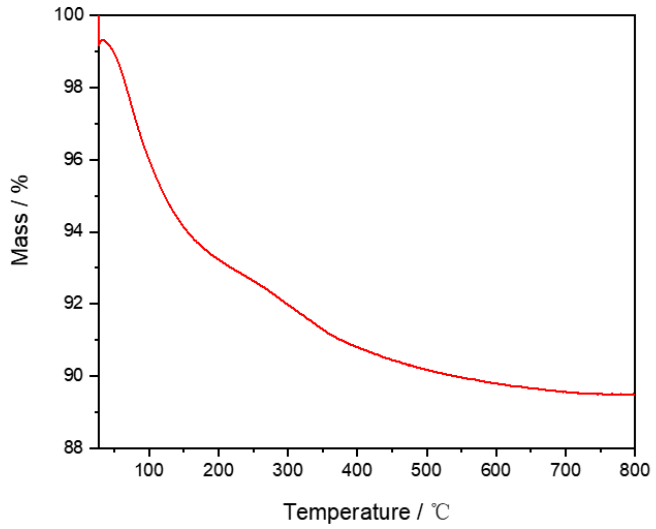

Figure S1. The thermogravimetric curve of the as-prepared product before high temperature calcination. The mass loss is found at a temperature lower than 400 °C, presumably due to dehydration at below 150 °C and dehydroxylation at 200 to 400 °C. The weight loss is not significant above 400 °C while a temperature as high as 700 °C is proved to be successful to transform high energy facets such as (100) and (110) to the thermodynamically stable (111) facets.[1] Thus a calcination temperature of 700 °C is used in the sample preparation process.

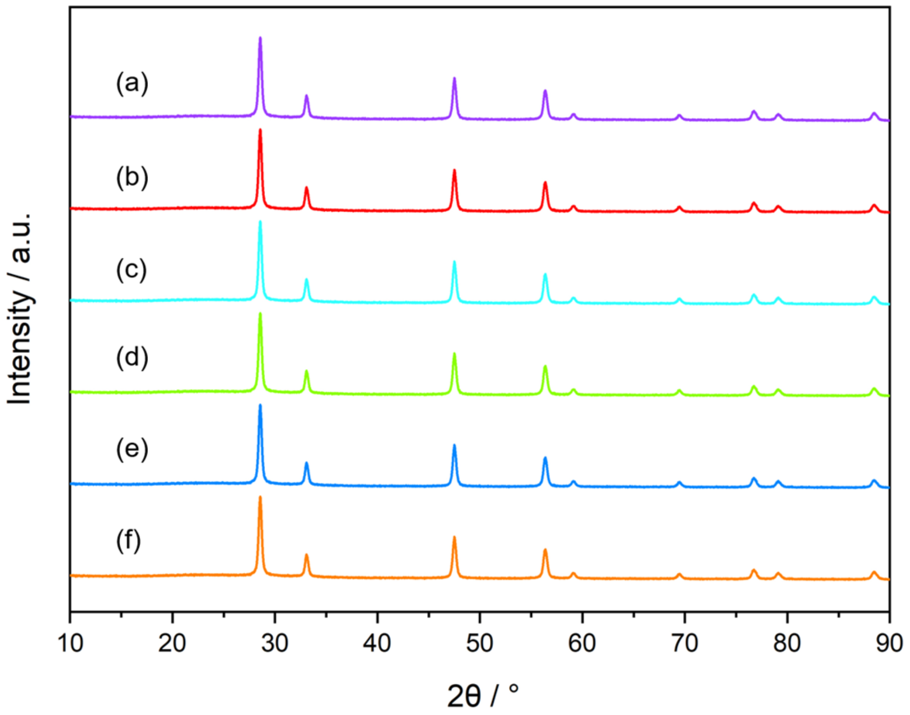

Figure S2. The XRD patterns of CeO_2 nanostructures obtained by pouring 0.05 mol·L^{-1} $CeCl_3$ solution to NaOH solutions with different concentrations. (a) 0.1 mol·L^{-1}, (b) 1 mol·L^{-1}, (c) 3 mol·L^{-1}, (d) 4 mol·L^{-1} (e) 5 mol·L^{-1} and (f) 6 mol·L^{-1}. Hydrothermal temperature: 100 °C, time: 24 h. Cerium source: 0.05 mol·L^{-1} $CeCl_3$ solution.

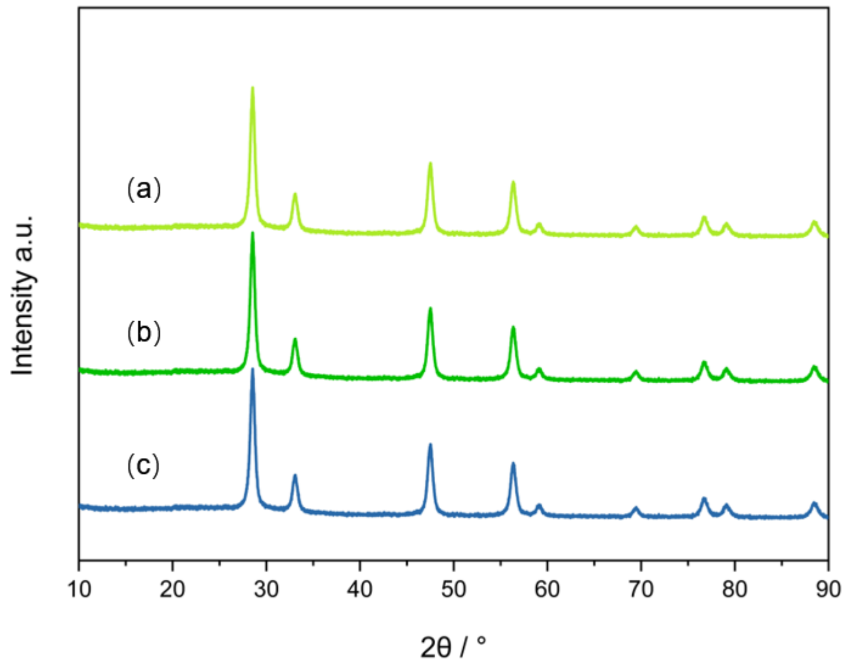

Figure S3. The XRD patterns of CeO_2 nanostructures obtained with different hydrothermal temperatures. (a) 100 ℃, (b) 140 ℃ and (c) 180 ℃. Hydrothermal time: 24 h. Cerium source: 0.05 mol·L^{-1} $CeCl_3$ solution. NaOH solution concentration: 6 mol·L^{-1}.

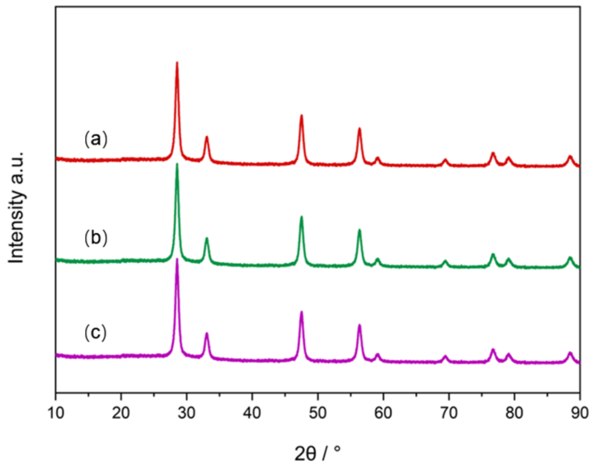

Figure S4. The XRD patterns of CeO_2 nanostructures obtained with different hydrothermal time. (a) 12 h, (b) 24 h and (c) 36 h. Hydrothermal temperature: 100 °C. Cerium source: 0.05 mol·L^{-1} $CeCl_3$ solution. NaOH solution concentration: 6 mol·L^{-1}.

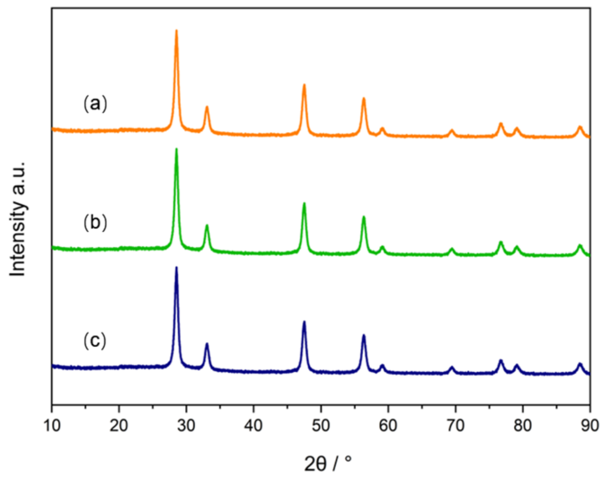

Figure S5. The XRD patterns of CeO_2 nanostructures obtained with different cerium sources. (a) $Ce(NO_3)_3$, (b) $CeCl_3$, (c) $Ce(NH_4)_2(NO_3)_6$. Hydrothermal temperature and time: 100 °C and 24 h. NaOH solution concentration: 6 $mol \cdot L^{-1}$.

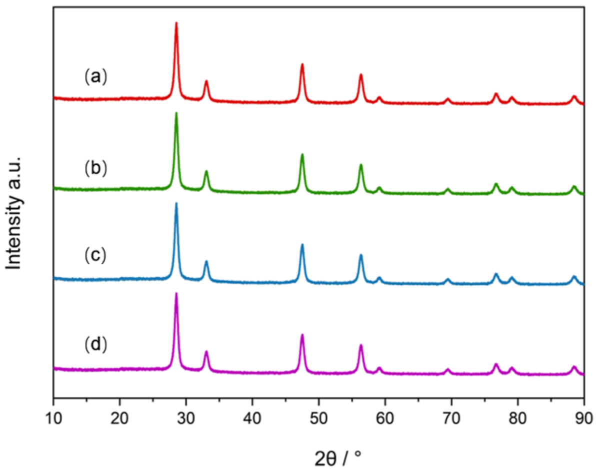

Figure S6. The XRD patterns of CeO_2 nanostructures obtained with different oxidants. (a) H_2O_2, (b) air (reaction time: 120 min), (c) air (reaction time: 30 min) and (d) trace oxygen in N_2 atmosphere. Hydrothermal temperature and time: 100 °C and 24 h. Cerium source: 0.05 mol·L^{-1} $CeCl_3$ solution. NaOH solution concentration: 6 mol·L^{-1}.

References

1. B. Sudduth, D. Yun, J. Sun and Y. Wang, *J. Catal.*, 2021, **404**, 96-108.
